# Artificial Intelligence (AI) driven patient assignment: optimizing daily redistribution among hospitalists

**DOI:** 10.3389/fmed.2026.1767258

**Published:** 2026-02-05

**Authors:** Dhaval Save, Nupoor Tillu

**Affiliations:** 1 Attending Physician, Internal Medicine, Carle Health Methodist Hospital, Peoria, IL, United States; 2 Attending Physician, Anesthesiology, Carle Health Methodist Hospital, Peoria, IL, United States

**Keywords:** artificial intelligence, hospital operations, hospitalists, patient assignment, patient redistribution

## 1 Introduction

In the ever-evolving healthcare landscape, Artificial Intelligence (AI) can revolutionize the patient distribution process among hospitalists. AI-driven patient redistribution (often called Automated Patient Assignment) is a rapidly growing field in hospital medicine. Historically, hospitalist “rounding lists” were created manually by a lead physician or administrator using a whiteboard or spreadsheet—a process that often took 2–3 h every morning. Today, AI systems accomplish this by directly integrating with Electronic Health Records (EHRs) to balance workloads in real-time. It has emerged as a transformative force in an otherwise manual, time-consuming process, bringing new efficiency and precision to healthcare delivery systems.

Hospitalists are physicians who specialize in the comprehensive care of hospitalized patients, managing everything from admission through discharge. They serve as the central coordinators of inpatient care, collaborating with specialists, nurses, and other healthcare professionals to ensure seamless treatment and optimal outcomes. Available around the clock within the hospital setting, hospitalists provide continuity and immediate responsiveness that traditional outpatient physicians cannot offer. Their expertise in managing acute medical conditions, navigating complex hospital systems, and facilitating efficient care transitions makes them indispensable to modern healthcare. By focusing exclusively on inpatient medicine, hospitalists have become the backbone of hospital operations, improving patient safety, reducing length of stay, and enhancing the overall quality of care delivery in an increasingly complex healthcare environment.

## 2 Present-day challenges

Daily patient distribution among hospitalists presents several complex operational hurdles. The process must balance competing factors, including individual physician workload caps, continuity of care preferences, geographic unit-based assignments, and varying patient acuity levels (1, 2). Hospitalists often have different experience levels and clinical interests that need to be considered, while some may have additional administrative or teaching responsibilities that affect their optimal patient load. The dynamic nature of admissions and discharges creates challenges in maintaining even distribution, potentially overwhelming some hospitalists while underutilizing others (3). The task is further complicated by the need to account for cross-coverage during off-hours, ensure appropriate hand-offs for patients requiring specialty consultation, and manage bounce-backs to avoid patient dissatisfaction (4). Weekend coverage and holiday scheduling add another layer of complexity, as does the need to factor in systemic issues such as language barriers between patients and providers or the geographic spread of patients across different hospital units (5).

## 3 How it works: understanding AI tools and methodology

**Figure 1:**
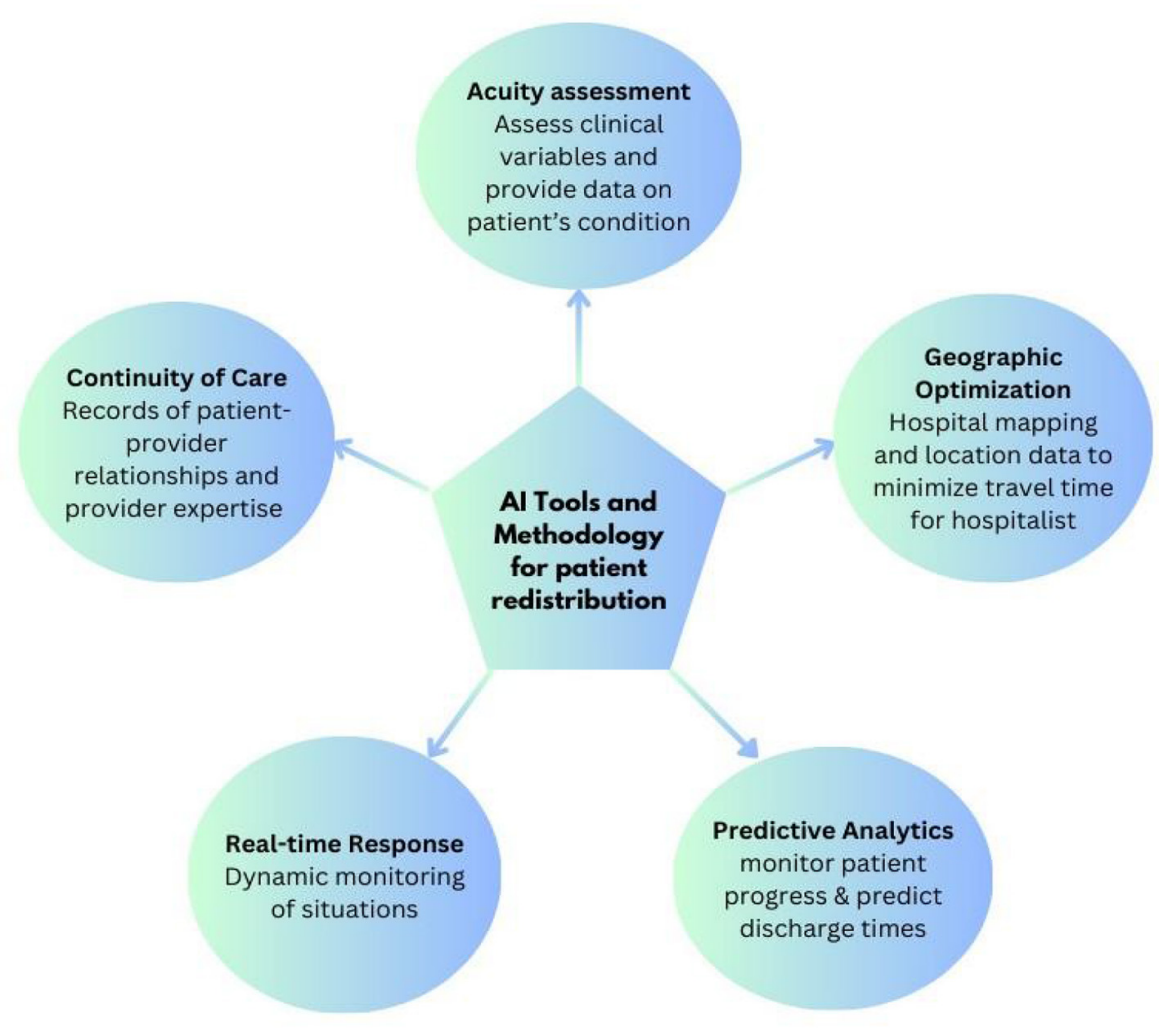
AI tools for patient redistribution.

The foundation of effective patient distribution lies in accurate workload assessment, where AI excels by processing multiple variables simultaneously [Figure 1].

### 3.1 Assessing patient acuity

Modern AI systems can analyze patient acuity levels with remarkable precision, accounting for basic metrics and the full complexity of care required for each case (6). These sophisticated systems continuously analyze several clinical variables in real-time, providing healthcare providers with crucial insights into patient conditions by processing vast amounts of data, including vital signs, laboratory results, medication responses, and nursing assessments (7).

### 3.2 Facility management

AI systems consider the geographic layout of hospital facilities, calculating optimal patient placement to minimize travel time for healthcare providers while maintaining high standards of care. These systems track real-time locations of patients and staff to determine optimal placement and provider assignments, while some focus on predictive bed management using pattern analysis (8). Advanced systems integrate with Real-Time Location Systems to accurately track resources and personnel, enabling workforce distribution across geographic zones. It can incorporate with the Hospital building management infrastructure to monitor occupancy, predict demand, and calculate optimal paths for healthcare providers across different units (9, 10).

### 3.3 Predictive analytics

By analyzing historical data patterns and current patient conditions, AI systems can accurately forecast likely discharge times (11). The system continuously monitors patient progress and updates predictions, providing a dynamic view of future hospital capacity and staffing needs. These predictive capabilities extend to anticipating potential transfers between units and estimating patient length of stay. By understanding these patterns, hospitals can better allocate resources and maintain optimal staffing levels across different departments. This proactive approach helps prevent bottlenecks in patient flow and ensures smoother transitions across care levels (12).

### 3.4 Real-time adaptation

Healthcare environments are inherently dynamic, with situations changing rapidly due to emergency admissions, unexpected discharges, or sudden changes in patient conditions (13). AI systems continuously monitor these changes and can recalculate optimal distribution patterns in real time, ensuring the workload remains balanced as conditions evolve (6). The system's ability to process multiple variables simultaneously allows it to suggest redistributions that account for both immediate needs and longer-term implications. When a change occurs, such as an emergency admission or an unexpected staff absence, the AI can quickly propose adjustments that maintain workload balance while minimizing disruption to existing care patterns (9).

### 3.5 Prioritizing care continuity

AI systems can strengthen patient-provider relationships by tracking interactions and matching patients with familiar providers when appropriate (10). The technology factors in language capabilities, cultural considerations, specialty expertise, and specific treatment requirements to ensure that patients receive care from the most appropriately qualified providers (14). Additionally, AI maintains detailed profiles of healthcare providers' specializations, past experiences, and strengths, enabling nuanced assignment decisions for complex cases and effective workload management for new hospitalists (15).

## 4 Implementation in current hospital systems & impact

Several major hospital systems have already moved past the pilot phase and fully integrated automated patient assignment and redistribution systems. Here are the specific systems, the technology they use, and the data-backed results they have reported [Table 1]:

**Table 1:** Examples of specific AI platforms currently leading the market.

**System**	**Primary focus**	**Notable features**
Medaptus (assign)	Workload balancing	Uses “configurable logic” to automate morning lists. It factors in census, acuity, and “continuity” rules.
Qventus	Capacity & flow	An AI “command center” that predicts discharge barriers and redistributes resources to prevent “boarding” in the ER.
Lightning bolt	Scheduling & equity	Uses “combinatorial optimization” to ensure shifts, night calls, and patient loads are equalized over months, not just days.
Ingenious med	Workflow & census	Features a “census manager” that allows for bulk drag-and-drop reassignment and real-time visibility of patient spikes.
Carealign	Task management	While not a pure “redistribution” engine, it acts as a digital “paper sheet” that syncs tasks across teams to prevent redundant work.

### 4.1 Northwell health (New York)

Northwell Health is one of the most prominent examples of success in this area. They implemented **Medaptus's “Assign”** software to replace manual spreadsheets and whiteboards. This reduced the lead hospitalists' morning assignment process time by 80% from 2.5 h down to about 30 min, saving roughly 2 h of administrative work per day for every lead physician, allowing them to start patient rounds significantly earlier and reducing the “morning bottleneck” of discharges (16).

### 4.2 HCA healthcare (National)

HCA, the largest for-profit hospital chain in the U.S., developed an internal AI-driven operational solution called **Timpani**. Although not used among physicians, this tool manages nursing shifts and patient loads and has shown its impact. The system monitors real-time needs on each nursing unit. If the AI detects a nurse is overloaded, or a patient is behind on scheduled care, it alerts clinical coordinators to rebalance patient loads mid-shift. HCA also uses AI for nurse handoffs, which they estimated previously took 40 min per shift. Early data from their pilot sites showed an 86% factuality rate in AI-generated reports, which significantly lightened the cognitive load on staff during redistribution (17).

### 4.3 Banner health (arizona/multistate)

Banner Health partnered with **Qventus**, a leading AI platform for hospital operations, to manage inpatient capacity and surgical scheduling. Across its facilities, Banner reported that the AI solution released 369 h of block time (operating room capacity) every month by predicting cancellations and reallocating time automatically. Qventus reported that its hospital clients (including Banner and Ardent Health) typically see a 15% to 30% reduction in “excess days” (days patients stay beyond what is medically necessary) and a 10% to 20% reduction in Emergency Department boarding times (18).

### 4.4 Pacific northwest health system

In a broader implementation across 12 hospitals, this system integrated AI-driven staff scheduling and patient load balancing. Within 6 months, the system reported about 32% reduction in nurse overtime, about 27% increase in staff satisfaction scores, and about 28% decrease in agency staffing costs (19).

## 5 Impact of AI on patient distribution

**Figure 2:**
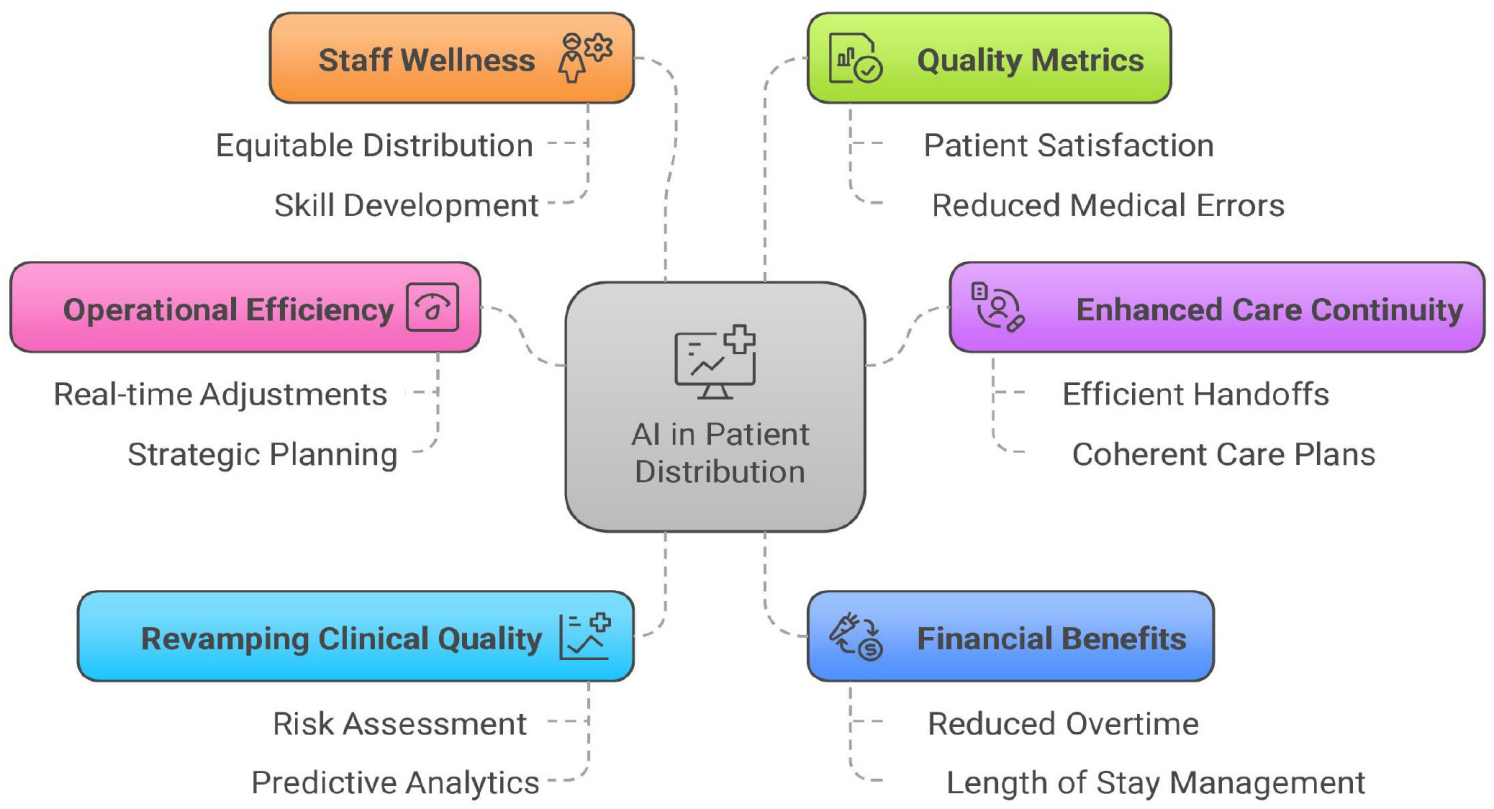
Impact of AI-driven patient redistribution.

Using AI to distribute patients among hospitalists represents a transformative advancement in healthcare operations management. It has a positive impact on the daily workflow in many ways [Figure 2].

### 5.1 Operational efficiency

Modern AI algorithms automate the previously manual process, freeing up significant time for hospitalist leaders and allowing them to focus on direct patient care and strategic planning. The system's ability to make real-time adjustments ensures dynamic redistribution when new admissions arrive or patient conditions change (20).

### 5.2 Enhanced care continuity

AI systems excel at maintaining consistent patient-physician relationships by tracking previous assignments and preferences. This systematic approach minimizes the number of different hospitalists seeing each patient, resulting in more efficient handoff management and more coherent care plans. The technology ensures that complex cases receive appropriate follow-up and consistent care throughout the patient's hospital stay (14).

### 5.3 Revamping clinical quality

AI's risk assessment capabilities enable precise matching of patient needs with hospitalist expertise. The system evaluates patient complexity and acuity to ensure equitable distribution of high-risk cases, while accounting for hospitalist subspecialty training and experience levels. Hospitals can anticipate patient flow patterns through merged predictive analytics and prepare proactively for admission surges or discharge patterns (15).

### 5.4 Financial benefits

There is a potential for substantial financial advantages through optimized resource utilization. Hospitals experience reduced overtime costs due to better workload balancing and decreased reliance on locum coverage. The system's efficient care coordination leads to appropriate length-of-stay management and reduced delays in care delivery, resulting in significant cost savings (21).

### 5.5 Staff wellness

AI-driven distribution systems promote staff wellbeing by ensuring equitable distribution of complex cases and patient load. The technology considers individual hospitalist preferences and scheduling constraints while providing opportunities for skill development through appropriate case mix assignments. This balanced approach significantly reduces the risk of burnout while enhancing professional growth opportunities (6).

### 5.6 Quality metrics

Patient satisfaction has markedly improved through enhanced continuity of care and reduced wait times. Consistent hospitalist assignments lead to better communication and patient experience. Clinical outcomes benefit from advanced management of complex cases and a reduced risk of medical errors, due to balanced workloads and consistent provider coverage (9).

## 6 Considerations and challenges

Successfully implementing AI-driven patient distribution requires careful planning and consideration of various factors. The technical infrastructure must be well-consolidated with existing hospital management systems. This merger should enable seamless data flow while maintaining high-security standards for patient and provider information.

### 6.1 Technical considerations

Blending existing Electronic Health Record systems must enable real-time access to patient data, including acuity scores, admission/discharge timing, and special care requirements. The AI system requires reliable interfaces with scheduling software and hospitalist management platforms (13).

### 6.2 Operational impact

Daily redistribution affects hospitalist workflows, handoffs, and continuity of care. Geographic clustering of patients' needs to be balanced against workload equity. The system must accommodate varying levels of hospitalist experience and specialties while maintaining appropriate patient-to-provider ratios (13).

### 6.3 Clinical safety

Patient safety depends on accurate handoff documentation and clear communication protocols during redistributions. The AI system must be robust enough to recognize complex cases that require continuity with specific providers and to account for hospitalists' familiarity with particular conditions or units (22).

### 6.4 Data requirements

The system needs comprehensive historical data on patient outcomes, hospitalist performance metrics, and workload patterns. Real-time inputs include fluctuations in census, admission patterns, and provider availability. Data quality and standardization across different hospital units are essential (20).

### 6.5 Implementation strategy

A phased rollout allows for system refinement and staff adaptation. Initial implementation should focus on essential load balancing before incorporating more complex factors. Regular evaluation of redistribution outcomes helps refine algorithms and identify needful adjustments (10).

### 6.6 Employee training

Success requires hospitalist buy-in through clear communication of benefits and addressing concerns about disrupted patient relationships. Training programs must prepare staff for new workflows and system interactions. Regular feedback channels help identify and resolve operational issues (23).

### 6.7 Cost implications

Implementation costs include software development, consolidation with existing systems, staff training, and ongoing maintenance. The Return On Investment (ROI) assessment should consider improved efficiency against potential increases in technical support needs (13).

### 6.8 Ethical/Legal considerations

Systems must be transparently designed, allowing healthcare administrators to understand the basis for distribution recommendations. Privacy protection and data security must be prioritized, and accountability structures should be established for system outcomes (24). The system must comply with privacy regulations and maintain audit trails of redistribution decisions. Clear policies should address liability concerns and establish override protocols when human judgment conflicts with AI recommendations (15). The goal is to support, not replace, human judgment in decisions, maintaining the critical role of experienced healthcare professionals in the distribution process.

## 7 Future outlook

As advanced analytics and machine learning capabilities continue to evolve, they will leverage increasingly larger datasets to improve prediction accuracy, employ sophisticated pattern recognition to optimize staffing models and resource allocation, and better integrate with telemedicine and cross-facility coordination (10). Future developments will include enhanced integration with hospital systems, improved team coordination tools, and advanced performance-monitoring capabilities. These technologies promise to significantly improve efficiency, provider satisfaction, and patient outcomes as healthcare organizations continue to refine their patient distribution processes.
